# Prevalence and factors associated with alcohol use disorders among people living with HIV attending care and treatment centers at Kilimanjaro, Tanzania: A cross-sectional study

**DOI:** 10.1371/journal.pone.0318120

**Published:** 2025-02-03

**Authors:** Florian Emanuel Ghaimo, Ester Steven Mzilangwe, Samuel Chacha, Saidi Bakari Kuganda

**Affiliations:** 1 Department of Psychiatry and Mental Health, Kilimanjaro Christian Medical University College, Moshi, Kilimanjaro, Tanzania; 2 Department of Psychiatry and Mental Health, Muhimbili University of Health and Allied Sciences, Dar es Salaam, Tanzania; 3 Department of Molecular Diagnostics, Sumbawanga Regional Referral Hospital, Rukwa, Tanzania; 4 Department of Psychiatry and Mental Health, Muhimbili National Hospital, Dar es Salaam, Tanzania; University of the Witwatersrand Johannesburg Faculty of Health Sciences, SOUTH AFRICA

## Abstract

**Background:**

Alcohol use disorders (AUD) are prevalent among people living with HIV (PLHIV), with 2–6 times higher than in the general population. These conditions are linked to increased morbidity and mortality among PLHIV and amplify sexual risk behaviors, thus exacerbating the transmission of HIV. Despite these negative consequences, a paucity of studies have explored this issue in Tanzania. This study aimed to determine AUD’s prevalence and associated factors among PLHIV attending Care and Treatment Centers (CTCs).

**Methods:**

A multifacility-based cross-sectional study was carried out among 532 PLHIV attending four CTC centers in Moshi Municipal, Kilimanjaro. A multistage cluster systematic sampling method was utilized to choose CTCs and participants. Data were collected using standardized tools through interviewer administration. Statistical analyses were performed using STATA (version 16). Binary logistic regression model was used to examine the associations between AUD and the independent variables, with odds ratios and their 95% confidence intervals calculated to quantify the strength of these associations.

**Results:**

The mean age of participants was 46.6 years (SD±13.3). The weighted prevalence of alcohol use disorders (AUDIT ≥ 8) within the past 12 months was 28.2%. Factors significantly associated with AUD in the final model included male sex (AOR = 4.18, P <0.001), healthcare level (reference: tertiary health facility; secondary health facility AOR = 1.80, P<0.001, primary health facility AOR = 9.65, P<0.001), being divorced or widowed (AOR = 2.82, P<0.001), secondary education (AOR = 1.35, P = 0.005), and probable depression (AOR = 2.48, P <0.001).

**Conclusion:**

The findings revealed a high prevalence of AUD among PLHIV, highlighting the need for policy refinement aimed at enhancing the integration of psychosocial services at CTCs.

## Introduction

HIV/AIDS has caused approximately 40.4 million fatalities since its discovery in the 1980s, making it one of the most lethal chronic health disorders globally [1]. In 2022, the number of HIV/AIDS-related deaths were approximately 630000, indicating its status as a significant public health issue [1]. The global HIV-positive population surpassed 39 million individuals, with the WHO African Region bearing the brunt approximately 1 in 25 adults (3.2%) are living with HIV, representing more than two-thirds of the global HIV-positive demographic [1]. In Tanzania, the national prevalence stands at 4.5%, accounting for approximately 1.7 million people living with HIV [2].

Globally, over 43% of individuals aged 15 and above use alcohol, and approximately 5.1% are affected by alcohol use disorders (AUD) [3]. AUD is linked to myriad health complications ranging from mental and behavioral problems to chronic conditions such as liver disease, cancers, and cardiovascular conditions. Moreover, AUD is associated with injuries following violence and road traffic accidents [4]. In 2016, AUD accounted for roughly 5.3% of all deaths across the globe and 5.1% of all disability-adjusted life years. Alcohol use causes more deaths than major diseases such as HIV/AIDS, TB, and diabetes [5].

Among the risk factors associated with increased morbidity and mortality among PLHIV, AUD plays a critical role [6–8]. Alcohol exacerbates immunosuppression, lessens cognitive function, hinders viral suppression, and directly associated with ART nonadherence, which can contribute to treatment failure and ART resistance [6, 9–11]. Furthermore, AUD encourages high-risk sexual behaviors, including unprotected sex, which in turn leads to further transmission of HIV [12–14]. Consequently, AUD poses a significant barrier to achieving the Joint United Nations Programme on HIV/AIDS (UNAIDS) targets, which aim for 95%-95%-95% of individuals to receive HIV testing and treatment, effectively suppressing the virus by 2025 [15].

Despite the adverse impacts of AUD on the health outcomes of PLHIV, it remains highly prevalent in this population [16], with 2–6 times higher than in the general population [17]. Factors such as young age, male sex, depressive disorders, family history of AUD, low education level, HIV-related stigma, employment status, marital status and limited social support have been identified as contributing factors to AUD among PLHIV [18–20].

Detecting and intervening in AUD at an early stage among PLHIV could enhance the efficacy of treatment, thereby playing a crucial role in reducing HIV/AIDS-related morbidity and mortality [18, 21]. Nevertheless, there is a paucity of published data from East Africa, particularly in Tanzania, on the magnitude and associated factors of AUD among PLHIV. This study aims to determine the prevalence and associated factors of AUD among PLHIV in Tanzania.

## Methods

### Study design and setting

A facility-based analytical cross-sectional study was carried out at four purposively chosen care and treatment centers (CTCs) based on health facilities levels located at Moshi, Kilimanjaro. The Moshi Municipal Council, encompassing approximately 184,292 residents and spanning 58 square kilometers, is one of seven districts in the Kilimanjaro region [22]. It serves approximately 800–1200 PLHIV a week across a total of 21 wards and 19 CTCs. The services offered at CTCs are integrated into all levels of healthcare facilities, from the lower primary level (health centers and dispensaries) to the secondary level (Mawenzi Regional Referral Hospital) and higher tertiary level (Kilimanjaro Christian Medical Center -KCMC Zonal Hospital). Kilimanjaro Christian Medical Center (KCMC) and Mawenzi Regional Referral Hospital (MRRH) were purposefully selected since they are the only tertiary and secondary-level health facilities, respectively. At the primary level, we selected Pasua and Majengo health centers because they both have high numbers of attendance compared to the remaining facilities in their category.

The eligibility criteria included adult PLHIV available during data collection, aged 18 years and above, receiving ART for at least 6 months, and provided informed consent to participate in the study. Participants too ill to participate or who withdrew during data collection were excluded. Approval for this study was obtained from the Muhimbili University of Health and Allied Sciences (MUHAS-REC-06-2023-1749), and permission for patient interviews was obtained from the regional administrative secretary and medical officer in charge of each study site. Participants were informed that those screening positive for probable AUD and clinically significant depressive episodes would be referred to appropriate mental health services.

### Sample size determination and sampling procedure

We estimated a minimum sample size of 532 using Cochran’s formula, referencing a previous Ethiopian study which reported AUD prevalence estimate of 31.8% [18]. Considering the involvement of multiple health facility levels (clusters), we adjusted for cluster variations by calculating the design effect utilizing the adjusted intracluster correlation coefficient that was used in the same region by Mushi *et al*. working on AUD in the general population, resulting in a sample size of 532 [23, 24]. We used proportional sampling to obtain the number of participants recruited from each clinic based on the number of patients seen at each clinic. To ensure sufficient representation of both sex in each healthcare facility, all male attendees were included in the sample, while a systematic sampling method was employed to select one of every three female attendees. This approach of oversampling men was implemented to balance the gender ratio, given that women outnumber men in healthcare attendance by an estimated ratio of 3:1. This sampling procedure has been shown to have good validity and reproducibility and has been used in several studies [23, 24].

### Data collection instruments and procedures

Data were collected by the principal investigator and four trained medical doctors who underwent one week of training. Interviewer-administered questionnaires utilizing REDCap software were implemented from September to October 2023. Tools assessing variables of interest are delineated below.

#### Demographic information

A sociodemographic questionnaire, developed by the researcher based on factors associated with AUD from previous studies, was employed to gather details on age, sex, education level, marital status, employment status, and family history of AUD. Medical records were also reviewed for the most recent viral load measurements.

#### Alcohol use disorders (AUD)

AUD was assessed using the Alcohol use disorders Identification Test (AUDIT). With the use of pictorial representations of various alcoholic beverages. Participants identified the kinds of alcoholic drinks they consumed (calculated based on a standard drink) that are commonly consumed in the Kilimanjaro region. This approach has been used in a previous study within the study region [25]. This study adopted the WHO’s definition of a standard alcoholic drink, which contains 10 grams of pure alcohol [26]. AUDIT is a fully structured questionnaire that has 10 elements totaling scores between 0 and 40 with different cut-offs, where scores < 8 indicates no AUD, while scores ≥ 8 denote the presence of AUD, which are further classified into three categories depending on the scores as follows: hazardous drinking (8–15), harmful drinking (16–19) and likely alcohol dependence (≥ 20). Although the AUDIT has not been specifically validated in Tanzania, it has been adapted from international studies across sub-Saharan nations, including Kenya, Mozambique, and Ethiopia [27, 28]. In Ethiopia, the Cronbach’s alpha for the AUDIT score was determined to be 0.9, with a sensitivity of 92% and a specificity of 87% [29]. It has been translated into the Swahili language and used in previous studies in northern Tanzania [24, 25].

#### Depression

We screened for probable depression using the Patient Health Questionnaire (PHQ-9), a prominent tool designed to screen for and assess the severity of depression. Developed by Kroenke *et al*. in 2001, it aligns with DSM-5 criteria by incorporating major depression symptoms into a concise, nine-item self-report questionnaire [30]. Each item asks respondents to rate how often they have experienced depressive symptoms over the past two weeks, with scores ranging from 0 to 3 (0 = not at all, 1 = several days, 2 = more than half the days, 3 = nearly every day). The total score can range from 0 to 27. Scores on the PHQ-9 are categorized as follows: 0–4 indicates no depression, 5–9 indicates mild depression, 10–14 indicates moderate depression, 15–19 indicates moderately severe depression, and 20–27 indicates severe depression. The PHQ-9 has been validated across different cultural contexts, including in Tanzania, where a Swahili version has been developed. Fawzi, *et al*. (2019) validated the PHQ-9 in Tanzania, reporting a Cronbach’s alpha of 0.83, indicating strong internal consistency [31]. In Tanzanian settings, a cutoff score of 9 has been suggested to indicate the presence of depression, while scores below 9 typically suggest the absence of significant depressive symptoms.

#### Perceived social support

We measured the level of perceived social support using the Duke-UNC Functional Social Support Questionnaire (FSSQ), which was originally designed for family medicine patients. It is often used as a self-administered evaluation tool and covers multiple dimensions of social support, including material, emotional, physical/instrumental, and social aspects. The 14 items are rated on a Likert scale ranging from 1 to 5, with a score of 5 representing total satisfaction with the level of support and a score of 1 representing total dissatisfaction. Higher scores, therefore, indicate greater social support, with a maximum score of 70. For this study, scores were categorized into tertiles: poor (14–23.3), fair/average (23.4–46.6), and good (46.7–70) [32]. The FSSQ has demonstrated good construct and concurrent validity; however, it shows questionable internal consistency, with a reported Cronbach’s alpha value of 0.66 [33], whereas a more recent study from 2013 showed very good reliability, with a value of 0.87 [34]. This tool has not been validated or adapted to the Tanzanian cultural context; however, it has been used by Madundo *et al*. on the same population of PLHIV in the same setting of northern Tanzania in a very recent study of 2023 assessing newly diagnosed depression [35].

#### HIV-related stigma

The HIV/AIDS stigma instrument (HASI-P) was utilized to assess HIV-related stigma. This 12-item scale evaluates personal stigma, self-image, disclosure, and public perception. It is assessed on a four-point scale from 1–4 (1 = strongly disagreed, 2 = disagreed, 3 = agreed, and 4 = strongly agreed that they have experienced HIV-related stigma since their diagnosis). Total scores range from 12 to 48, with higher scores indicating greater anticipated stigma. The HASI-P has been validated in several African countries, including Lesotho, Malawi, South Africa, Swaziland, and Tanzania, and it was found to have a Cronbach’s alpha >0.7, with a cutoff score of ≥ 30 indicating high perceived HIV stigma and a score of < 30 reflecting low perceived HIV stigma [36]. It has been utilized by Gamassa *et al*. on the same population of PLHIV in the same setting of northern Tanzania in a relatively recent 2023 study that addressed the problem of depression and suicidal ideation [37].

### Data processing and analyses

Data were extracted from REDCap software and analyzed using STATA version 16. Categorical variables were summarized using frequencies and percentages, while continuous variables were described using means and standard deviations. The results are presented in tables and figures. Bivariate and multivariate logistic regression models were performed to determine associations between independent and outcome variables. These models were adjusted for sampling weights and clustering by site to account for unequal selection probabilities and design effects. Odds ratios, along with their corresponding 95% confidence intervals, were calculated to assess the associations between the independent variables and dependent variable (AUD) with a significance level set at p<0.05.

## Results

### Sociodemographic and clinical characteristics of the participants

A total of 543 participants were initially recruited for the study. After excluding 11 participants (7 who opted out and 4 who had been on ART for less than 6 months), 532 participants were included. The mean age of the participants was 46.6 years (SD ± 13.3). Over half (56.4%) were aged between 36 and 59 years, and the majority (71.4%) were female. Regarding healthcare access, 43.4% received care at the secondary healthcare level (Mawenzi Regional Referral Hospital). In terms of marital status, 41% were married or cohabiting, 36.8% were divorced or widowed, and 22.2% had never married. About two-thirds (64.7%) of participants had primary level of education, and 76.7% were either formally employed or self-employed. Nearly one-third (32.1%) had detectable viral loads, and 27.1% reported a positive family history of AUD. Additionally, over two-thirds (66.4%) reported poor perceived social support, 14.8% screened positive for probable depression, and 71.6% experienced a high level of perceived HIV stigma. (Table 1).

**Table 1:** Sociodemographic and clinical characteristics of the participants (n = 532).

Characteristics	N (%)
**Age (years)**	Mean 46.6, SD 13.3
18–35	141(26.5)
36–59	300 (56.4)
≥60	91 (17.1)
**Sex**	
Female	380 (71.4)
Male	152 (28.6)
**Level of health facility**	
Tertiary health facility	157 (29.5)
Secondary health facility	231 (43.4)
Primary health facility	144 (27.1)
**Marital status**	
Single/never married	118 (22.2)
Married/Cohabiting	218 (41.0)
Divorced/Widowhood	196 (36.8)
**Education level**	
None	20 (3.8)
Primary	344 (64.7)
Secondary	136 (25.6)
College/Higher	32 (5.9)
**Current occupation**	
Unemployed	124 (23.3)
Employed	408 (76.7)
**Recent viral load count**	
Not detected (<20 copies/ml)	361 (67.9)
Detected (≥ 20 copies/ml)	171 (32.1)
**Family history AUD**	
No	388 (72.9)
Yes	144 (27.1)
**Level of social support**	
Poor (14–23.3)	353 (66.4)
Average (23.4–46.6)	129 (24.3)
Good (46.7–70)	50 (9.3)
**Probable depression**	
No (score < 9)	453 (85.2)
Yes (score ≥9)	79 (14.8)
**Level of perceived HIV stigma**	
High (score ≥30)	381 (71.6)
Low (score <30)	151 (28.4)

### Prevalence of alcohol use disorders

**Fig 1:**
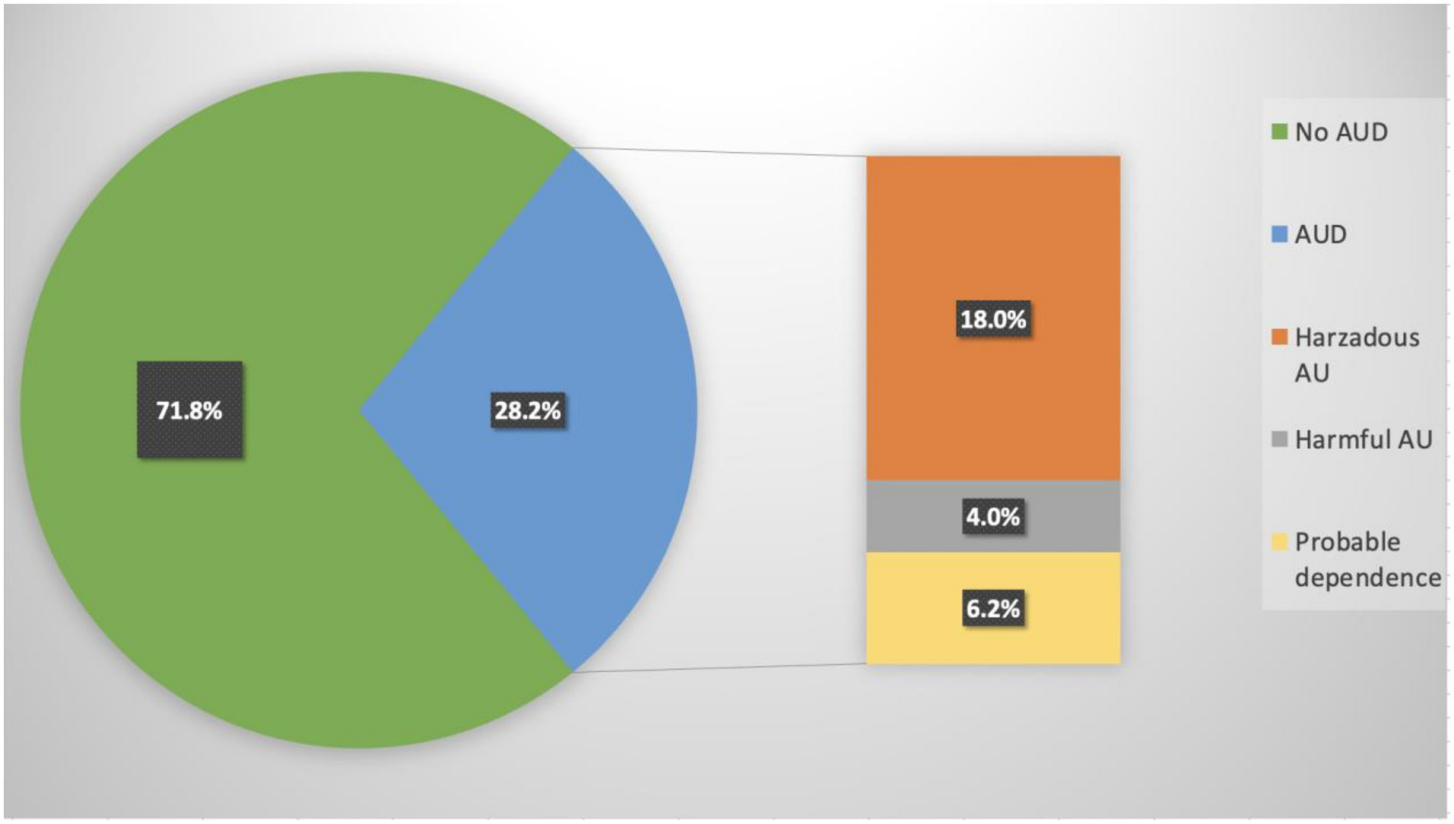
Prevalence of alcohol use disorders among people living with HIV at Moshi Municipality, Kilimanjaro, Tanzania.

Overall, slightly more than half of participants (52.8%) of the participants reported using alcohol within the past 12 months, with nearly one-third (29.7%) reporting heavy episodic drinking. The weighted prevalence of AUD (AUDIT ≥ 8) was 28.2%. Among participants who reported alcohol use in the past year, 18.0% had hazardous alcohol use (AUDIT score 8–15), 4.0% had harmful alcohol use (AUDIT score 16–19), and 6.2% had probable alcohol dependence (AUDIT score ≥ 20) as depicted in Fig 1.

### Factors associated with alcohol use disorders

The factors significantly associated with AUD in the final multivariate analysis were sex, marital status, education level, probable depression, and health facility status.

Sex was identified as an associated factor for AUD, with male participants being four times more likely to have AUD than females (AOR = 4.18, 95% CI: 3.84–4.54), and participants who were divorced or widowed were three times more likely to have AUD than those who were never married (AOR = 2.82, 95% CI: 1.58–5.04, P<0.001). Participants who had at least a secondary level of education had a 35% greater likelihood of having AUD than those who with no formal education or only primary education (AOR = 1.35, 95% CI: 1.09–1.68, P = 0.005).

Additionally, participants who screened positive for probable depression had a twofold increased likelihood of AUD compared to those screened negative (AOR = 2.48, 95% CI: 2.15–2.86). Notably, participants attending primary health facilities were nearly ten times more likely to have AUD than those attending tertiary health facilities (AOR = 9.65, 95% CI: 7.64–12.18, P<0.001), while participants attending secondary health facilities were nearly two times more likely to have AUD than those attending tertiary health facilities (AOR = 1.80, 95% CI: 1.68–1.93, P<0.001). (Table 2).

**Table 2:** Factors associated with AUD among PLHIV attending CTC centers at Moshi Municipal, Kilimanjaro.

Variable	Total	Reported AUD	COR (95% CI)	P	AOR (95% CI)	Adj.P
**Age (years)**						
18–35	141	27 (19.2)	Ref		Ref	
36–59	300	74 (24.7)	1.39 (1.28–1.50)	<0.001*	1.06 (0.81–1.39)	0.680
≥60	91	22 (24.2)	1.48 (0.44–4.99)	0.528	1.22 (0.31–4.83)	0.773
**Sex**						
Female	380	63 (16.6)	Ref		Ref	
Male	152	60 (39.5)	3.48 (2.74–4.40)	<0.001*	4.18 (3.84–4.54)	<0.001*
**Level of health facility**						
Tertiary health facility	157	17 (10.8)	Ref		Ref	
Secondary health facility	231	39 (16.9)	1.67 (1.67–1.67)	<0.001*	1.80 (1.68–1.93)	<0.001*
Primary health facility	144	67 (46.5)	7.17 (7.17–7.17)	<0.001*	9.65 (7.64–12.18)	<0.001*
**Marital status**						
Single/never married	118	20 (17.0)	Ref		Ref	
Married/Cohabiting	218	45 (20.6)	1.55 (0.98–2.46)	0.063	1.52 (0.56–4.19)	0.411
Divorced/Widowhood	196	58 (29.6)	2.25 (1.68–3.00)	<0.001*	2.82 (1.58–5.04)	<0.001*
**Education level**						
Informal/primary	364	82 (22.5)	Ref		Ref	
Secondary	136	33(24.3)	1.15 (0.97–1.37)	0.115	1.35 (1.09–1.68)	0.005*
College/Higher	32	8(25.0)	1.72 (0.41–7.26)	0.462	3.47 (0.56–21.37)	0.179
**Current occupation**						
Unemployed	124	30 (24.2)	Ref		Ref	
Employed	408	93 (22.8)	0.90 (0.69–1.18)	0.440	1.01 (0.76–1.45)	0.930
**Recent viral load count**						
Not detected	361	78 (21.6)	Ref		Ref	
Detected	171	45 (26.3)	1.33 (1.20–1.48)	<0.001*	1.34 (0.92–1.96)	0.129
**Family history of AUD**						
No	388	78 (20.1)	Ref		Ref	
Yes	144	45 (31.3)	2.04 (1.39–2.98)	<0.001*	1.76 (0.98–3.15)	0.058
**Level of social support**						
Good	50	17 (34.0)	Ref		Ref	
Average	129	37 (28.7)	0.78 (0.64–0.96)	<0.017*	0.90 (0.42–1.28)	0.795
Poor	353	69 (19.6)	0.47 (0.29–0.77)	<0.003*	0.55 (0.22–1.34)	0.186
**Probable depression**						
No	453	91 (20.1)	Ref		Ref	
Yes	79	32 (40.5)	3.07 (1.98–4.76)	<0.001*	2.48 (2.15–2.86)	<0.001*
**Level of perceived HIV stigma**						
Low	151	35 (23.1)	Ref		Ref	
High	381	381(23.2)	1.18 (0.66–2.11)	0.573	1.05 (0.44–2.50)	0.927

* Significant association (p-value < 0.05), COR- Crude odds ratio, AOR- Adjusted odds ratio

## Discussion

AUD is recognized as one of the prevalent mental health conditions associated with HIV, and it is linked to increased morbidity and mortality in this population. The present study represents one of the first studies aimed at determining the prevalence and factors associated with AUD among adult PLHIV attending CTCs at Moshi Municipal, Tanzania. Our findings indicate a high prevalence of AUD, with significant associations identified for male sex, treatment at lower-level health facilities, being widowed or divorced, having at least a secondary education, and screening positive for probable depression.

The prevalence of AUD among PLHIV in the past 12 months in this study using the AUDIT tool with a cutoff of ≥8 was 28.20%. This is consistent with findings from previous studies conducted both globally and locally. A systematic review and meta-analysis that was performed in both high- and low-middle-income countries, revealed that the pooled prevalence of AUD among PLHIV was 29.80% [38]. Similar findings were also replicated in studies from middle-income countries such as Brazil and Vietnam, which reported that the prevalence of AUD among PLHIV utilizing the AUDIT tool was 28.6% and 30.1%, respectively [16, 39]. Studies from sub-Saharan African countries also echoed these findings, with Ethiopian, Tanzanian, Ugandan, and South African studies reporting prevalence rates of 31.80%, 29.3%, 27.70%, and 28.77%, respectively [18, 38, 40, 41]. Conversely, some studies from high-income countries have reported higher prevalence of AUD. For instance, a pooled prevalence of 42.09% was revealed in a systematic review of high-income countries, and a pooled prevalence of 62% was reported in a Russian study [19, 38]. These discrepancies may be attributed to socioeconomic factors, as individuals in high-income countries often have greater financial means to purchase alcohol. Additionally, cultural norms surrounding alcohol consumption, such as the tradition of drinking alcohol with meals in high-income countries, may also contribute to these variation [3].

In our study, male sex, being widowed or divorced, and having at least a secondary education, were the identified sociodemographic factors that increased the likelihood of having AUD. These findings echo results from numerous studies across the globe as well as from sub-Saharan Africa [24, 25, 40]. The increased likelihood of AUD in men may be explained by neurochemical differences, with men exhibiting more robust dopamine release in response to alcohol than women despite consuming similar levels of alcohol, which may enhance alcohol-seeking behaviors [42]. Widowed individuals report worse mental health and more depressive symptoms than never-married and married people, which may drive increased substance use as a coping mechanism [43]. Contrary to some previous studies linking lower education levels with AUD [16], our findings indicate that attaining at least a secondary level of education increases the likelihood of AUD. This finding may be explained by greater awareness among individuals with higher education regarding the minimal direct interactions between ARV and alcohol [44], potentially encouraging to increased alcohol use.

Additionally, participants who screened positive for depression had an increased likelihood of having AUD compared to their counterparts. This finding may be explained by the fact that patients with long-term medical conditions, such as HIV, frequently have to change their life objectives, aspirations, and lifestyles owing to their medical conditions, which causes emotional suffering and may eventually make them more susceptible to depression and hence consuming alcohol as a coping mechanism and to self-medicate their depressive symptoms [44, 45].

Furthermore, participants attending lower levels of health facilities, such as primary and secondary health care facilities exhibited a higher likelihood of AUD compared to those accessing tertiary facilities. This may be influenced by access to psychosocial interventions in tertiary facilities, including brief interventions such as psychoeducation and motivational interviewing about alcohol use, which are integrated into its CTCs [46]. Additionally, specialized mental health services are available for severe cases of AUD, potentially resulting in a lower likelihood of AUD compared to participants attending primary and secondary health facilities where these services are limited [46].

Despite a substantial proportion of participants in the present study reporting poor level of social support and a high level of perceived HIV stigma, their association with AUD was not statistically significant. This contrasts with findings from some studies that suggest inadequate social support and high levels of HIV-related stigma may predispose individuals to AUD as a coping mechanism. A Russian study, conducted in a country with the highest number of PLHIV in Europe, found an increased likelihood of AUD among PLHIV with poor level of social support, as measured by the Social Provisions Scale (SPS) [19]. Similarly, a sub-Saharan study in Ethiopia, using the Oslo 3-item Social Support Scale, reported comparable findings among PLHIV [18]. Furthermore, high levels of HIV-related stigma were found to increase the likelihood of AUD in studies from Europe and South Africa [19, 47].

These discrepancies may be partially due to differences in the measurement tools used. The current study employed the Duke-UNC Functional Social Support Questionnaire (FSSQ) to assess social support, while the Russian and Ethiopian studies used the Social Provisions Scale (SPS) and the Oslo 3-item Social Support Scale, respectively. For measuring HIV stigma, the present study used the HIV/AIDS Stigma Instrument (HASI-P), while the other studies utilized a sum of scores across two enacted stigma and two anticipated stigma items [47].

## Recommendations

These findings underscore a pressing necessity for the immediate action of multidisciplinary stakeholders to refine policies to advocate for the enhancement of integration of psychosocial services such as screening and providing brief interventions for common mental disorders such as AUD at the CTCs in all levels of health care, as well as the referral of severe cases to higher levels of health facilities for specialized care as stipulated in recent Tanzania guidelines for the management of HIV/AIDS, and the WHO Mental Health Gap Action Program (MhGap) for mental, neurological and substance abuse (MNS). Additionally, further research to explore the barriers to implementation and efficiency of these interventions at CTCs is also crucial.

## Limitations

This study’s findings should be interpreted with awareness of several limitations. Participant bias could have occurred due to participants’ potential social desirability to select which information to communicate due to the issues of stigma around HIV and alcohol use in the community, which could have underestimated or overestimated the results. Additionally, the Duke-UNC Functional Social Support Questionnaire tool employed in this study has not been validated in the Tanzania context, which may impact the accuracy of our findings. Despite these limitations, this research successfully achieved the targeted sample size and included participants from various levels of healthcare facilities spanning primary to tertiary care, enhancing the generalizability of the results.

## Conclusion

This study reveals nearly one in three PLHIV has AUD, a finding consistent with many studies in low- to middle-income countries. Being male, treatment at primary and secondary health facilities, being divorced or widowed, having at least a secondary education, and having probable depression were associated with having probable AUD. These results highlight the necessity for policy refinement to enhance the integration of psychosocial services into HIV care and treatment clinics to facilitate the timely detection and management of AUD.

## Supporting information

S1 File (XLS)

## References

[1] Joint United Nations Programme on HIV/AIDS (UNAIDS). Fact sheet 2024—Latest global and regional HIV statistics on the status of the AIDS epidemic. 2024; https://www.unaids.org/en

[2] UNAIDS. HIV-AIDS Tanzania Factsheet. 2023(UNAIDS 2021):1–3. https://www.usaid.gov/tanzania/fact-sheet/jun-13-2023-tanzania-hivaids-fact-sheet

[3] WHO. Global alcohol action plan [Internet]. 2022. https://iris.who.int/bitstream/handle/10665/376939/9789240090101-eng.pdf?sequence=1

[4] Organização Mundial de Saúde. World health statistics 2022 (Monitoring health of the SDGs) [Internet]. Monitoring health of the SDGs. 2022. 1–131 p. http://apps.who.int/bookorders.

[5] Hammer JH, Parent MC, Spiker DA. Mental Help Seeking Attitudes Scale (MHSAS): Development, reliability, validity, and comparison with the ATSPPH-SF and IASMHS-PO. Journal of counseling psychology. 2018 Jan;65(1):74. doi: 10.1037/cou0000248 29355346 PMC10460514

[6] Akgün KM, Gordon K, Pisani M, Fried T, McGinnis KA, Tate JP, et al. Risk factors for hospitalization and medical intensive care unit (MICU) admission among HIV-infected Veterans. JAIDS Journal of Acquired Immune Deficiency Syndromes. 2013 Jan 1;62(1):52–9. doi: 10.1097/QAI.0b013e318278f3fa 23111572 PMC4182723

[7] Azar MM, Springer SA, Meyer JP, Altice FL. A systematic review of the impact of alcohol use disorders on HIV treatment outcomes, adherence to antiretroviral therapy and health care utilization. Drug and alcohol dependence. 2010 Dec 1;112(3):178–93. doi: 10.1016/j.drugalcdep.2010.06.014 20705402 PMC2997193

[8] Crawford TN, Thornton AC. Alcohol use and multimorbidity among individuals living with HIV. AIDS and Behavior. 2019 Jan 15;23:152–60. doi: 10.1007/s10461-018-2242-y 30088200

[9] Velloza J, Kemp CG, Aunon FM, Ramaiya MK, Creegan E, Simoni JM. Alcohol use and antiretroviral therapy non-adherence among adults living with HIV/AIDS in sub-Saharan Africa: a systematic review and meta-analysis. AIDS and Behavior. 2020 Jun;24:1727–42. doi: 10.1007/s10461-019-02716-0 31673913 PMC7190427

[10] Pokhrel KN, Gaulee Pokhrel K, Neupane SR, Sharma VD. Harmful alcohol drinking among HIV-positive people in Nepal: an overlooked threat to anti-retroviral therapy adherence and health-related quality of life. Global health action. 2018 Jan 1;11(1):1441783. doi: 10.1080/16549716.2018.1441783 29495948 PMC5844022

[11] Baum MK, Rafie C, Lai S, Sales S, Page JB, Campa A. Alcohol use accelerates HIV disease progression. AIDS research and human retroviruses. 2010 May 1;26(5):511–8. doi: 10.1089/aid.2009.0211 20455765 PMC2875959

[12] Avalos LA, Mertens JR, Ward CL, Flisher AJ, Bresick GF, Weisner CM. Stress, substance use and sexual risk behaviors among primary care patients in Cape Town, South Africa. AIDS and Behavior. 2010 Apr;14:359–70. doi: 10.1007/s10461-009-9525-2 19205865 PMC2835823

[13] Shuper PA, Joharchi N, Irving H, Rehm J. Alcohol as a correlate of unprotected sexual behavior among people living with HIV/AIDS: review and meta-analysis. AIDS and Behavior. 2009 Dec;13:1021–36. doi: 10.1007/s10461-009-9589-z 19618261

[14] Gerbi GB, Habtemariam T, Tameru B, Nganwa D, Robnett V. The correlation between alcohol consumption and risky sexual behaviours among people living with HIV/AIDS. Journal of substance use. 2009 Jan 1;14(2):90–100. 19693283 10.1080/14659890802624261 PMC2728293

[15] Heath K, Levi J, Hill A. The Joint United Nations Programme on HIV/AIDS 95–95–95 targets: worldwide clinical and cost benefits of generic manufacture. Aids. 2021 Dec 15;35(Supplement 2):S197–203. doi: 10.1097/QAD.0000000000002983 34115649

[16] Da Silva CM, Mendoza-Sassi RA, da Mota LD, Nader MM, de Martinez AM. Alcohol use disorders among people living with HIV/AIDS in Southern Brazil: prevalence, risk factors and biological markers outcomes. BMC infectious diseases. 2017 Dec;17:1–8. 28399823 10.1186/s12879-017-2374-0 PMC5387222

[17] Galvan FH, Bing EG, Fleishman JA, London AS, Caetano R, et al. The prevalence of alcohol consumption and heavy drinking among people with HIV in the United States: results from the HIV Cost and Services Utilization Study. Journal of studies on alcohol. 2002 Mar;63(2):179–86. doi: 10.15288/jsa.2002.63.179 12033694

[18] Duko B, Toma A, Abraham Y. Alcohol use disorder and associated factors among individuals living with HIV in Hawassa City, Ethiopia: a facility based cross-sectional study. Substance abuse treatment, prevention, and policy. 2019 Dec;14:1–6. 31109353 10.1186/s13011-019-0212-7 PMC6528325

[19] Lunze K, Lioznov D, Cheng DM, Nikitin RV, Coleman SM, et al. HIV stigma and unhealthy alcohol use among people living with HIV in Russia. AIDS and Behavior. 2017 Sep;21:2609–17. doi: 10.1007/s10461-017-1820-8 28600603 PMC5709173

[20] Lipira L, Rao D, Nevin PE, Kemp CG, Cohn SE, Turan JM, et al. Patterns of alcohol use and associated characteristics and HIV-related outcomes among a sample of African-American women living with HIV. Drug and alcohol dependence. 2020 Jan 1;206:107753. doi: 10.1016/j.drugalcdep.2019.107753 31785536 PMC6980681

[21] Koyra HC. Adherence to antiretroviral therapy among adult persons living with HIV/AIDS in Southern Ethiopia. Int J Virol AIDS. 2018;5(038):10–23937.

[22] NBS (National Bureau of Statistics). Tanzania in Figures 2012.

[23] Adams G, Gulliford MC, Ukoumunne OC, Eldridge S, Chinn S, Campbell MJ. Patterns of intra-cluster correlation from primary care research to inform study design and analysis. Journal of clinical epidemiology. 2004 Aug 1;57(8):785–94. doi: 10.1016/j.jclinepi.2003.12.013 15485730

[24] Mushi D, Moshiro C, Hanlon C, Francis JM, Teferra S. Missed opportunity for alcohol use disorder screening and management in primary health care facilities in northern rural Tanzania: a cross-sectional survey. Substance Abuse Treatment, Prevention, and Policy. 2022 Jul 6;17(1):50. doi: 10.1186/s13011-022-00479-x 35794580 PMC9258127

[25] Francis JM, Weiss HA, Mshana G, Baisley K, Grosskurth H, Kapiga SH. The epidemiology of alcohol use and alcohol use disorders among young people in northern Tanzania. PloS one. 2015 Oct 7;10(10):e0140041. doi: 10.1371/journal.pone.0140041 26444441 PMC4596556

[26] Stockwell T, Chikritzhs T, Holder H, Single E, Elena M, Jernigan D. International guide for monitoring alcohol consumption and related harm. Geneva, Switzerland: World Health Organization. 2000:1Á193.

[27] Atkins DL, Cumbe VF, Muanido A, Manaca N, Fumo H, et al. Validity and item response theory properties of the Alcohol Use Disorders Identification Test for primary care alcohol use screening in Mozambique (AUDIT-MZ). Journal of substance abuse treatment. 2021 Aug 1;127:108441. doi: 10.1016/j.jsat.2021.108441 34134876 PMC8217722

[28] Habtamu E, Madoro D. Psychometric properties of alcohol use disorder Identification test screening tool among medical outpatients in dilla university referral hospital, southern Ethiopia, 2020. SAGE Open Medicine. 2022 Feb;10:20503121221077568. doi: 10.1177/20503121221077568 35186293 PMC8855433

[29] Saunders JB, Aasland OG, Babor TF, De la Fuente JR, Grant M. Development of the alcohol use disorders identification test (AUDIT): WHO collaborative project on early detection of persons with harmful alcohol consumption-II. Addiction. 1993 Jun;88(6):791–804. doi: 10.1111/j.1360-0443.1993.tb02093.x 8329970

[30] Kroenke K, Spitzer RL, Williams JB. The PHQ‐9: validity of a brief depression severity measure. Journal of general internal medicine. 2001 Sep;16(9):606–13. doi: 10.1046/j.1525-1497.2001.016009606.x 11556941 PMC1495268

[31] Fawzi MC, Ngakongwa F, Liu Y, Rutayuga T, Siril H, et al. Validating the Patient Health Questionnaire-9 (PHQ-9) for screening of depression in Tanzania. Neurology, Psychiatry and Brain Research. 2019 Feb 1;31:9–14. doi: 10.1016/j.npbr.2018.11.002 32863596 PMC7455004

[32] Ziaei S, Contreras M, Blandón EZ, Persson LÅ, Hjern A, Ekström EC. Women’s autonomy and social support and their associations with infant and young child feeding and nutritional status: community-based survey in rural Nicaragua. Public health nutrition. 2015 Aug;18(11):1979–90. doi: 10.1017/S1368980014002468 25409706 PMC10271509

[33] Broadhead WE, Gehlbach SH, De Gruy FV, Kaplan BH. The Duke-UNC Functional Social Support Questionnaire: Measurement of social support in family medicine patients. Medical care. 1988 Jul 1:709–23. 10.1097/00005650-198807000-00006 3393031

[34] Mas-Expósito L, Amador-Campos JA, Gómez-Benito J, Lalucat-Jo L. Validation of the modified DUKE-UNC Functional Social Support Questionnaire in patients with schizophrenia. Social psychiatry and psychiatric epidemiology. 2013 Oct;48:1675–85. doi: 10.1007/s00127-012-0633-3 23229203

[35] Madundo K, Knettel BA, Knippler E, Mbwambo J. Prevalence, severity, and associated factors of depression in newly diagnosed people living with HIV in Kilimanjaro, Tanzania: a cross-sectional study. BMC psychiatry. 2023 Feb 1;23(1):83. doi: 10.1186/s12888-022-04496-9 36726113 PMC9890688

[36] Holzemer WL, Uys LR, Chirwa ML, Greeff M, Makoae LN, et al. Validation of the HIV/AIDS stigma instrument—Plwa (Hasi-P). AIDS care. 2007 Sep 1;19(8):1002–12. doi: 10.1080/09540120701245999 17851997

[37] Gamassa E, Steven E, Mtei R, Kaaya S. Prevalence of depression and suicidal ideation and associated risk factors in adolescents receiving care and treatment for HIV/AIDS at a tertiary health facility in Kilimanjaro Region, Tanzania. Research Square. [Preprint]. 2023 Feb 28:rs.3.rs-2534893. [Version 1] doi: 10.21203/rs.3.rs-2534893/v1 36909487 PMC10002847

[38] Duko B, Ayalew M, Ayano G. The prevalence of alcohol use disorders among people living with HIV/AIDS: a systematic review and meta-analysis. Substance abuse treatment, prevention, and policy. 2019 Dec;14:1–9. 31727086 10.1186/s13011-019-0240-3 PMC6854786

[39] Hershow RB, Zuskov DS, Vu Tuyet Mai N, Chander G, Hutton HE, et al. “[Drinking is] Like a Rule That You Can’t Break”: perceived barriers and facilitators to reduce alcohol use and improve antiretroviral treatment adherence among people living with HIV and alcohol use disorder in Vietnam. Substance use & misuse. 2018 Jun 7;53(7):1084–92. doi: 10.1080/10826084.2017.1392986 29537932 PMC6198809

[40] Ayieko P, Kisanga E, Mshana G, Nkosi S, Hansen CH, et al. Epidemiology of alcohol use and alcohol use disorders among people living with HIV on antiretroviral therapy in Northwest Tanzania: implications for ART adherence and case management. AIDS care. 2024 May 3;36(5):652–60. doi: 10.1080/09540121.2023.2299324 38295268

[41] Patts GJ, Cheng DM, Bridden C, Gnatienko N, Lloyd-Travaglini CA, et al. Alcohol use and food insecurity among people living with HIV in Mbarara, Uganda and St. Petersburg, Russia. AIDS and Behavior. 2017 Mar;21:724–33. doi: 10.1007/s10461-016-1556-x 27699595 PMC5303539

[42] Necho M, Belete A, Getachew Y. The prevalence and factors associated with alcohol use disorder among people living with HIV/AIDS in Africa: a systematic review and meta-analysis. Substance abuse treatment, prevention, and policy. 2020 Dec;15:1–5. 32831129 10.1186/s13011-020-00301-6 PMC7444054

[43] Yimer YM, Buli MB, Nenko G, Mirkena Y, Kassew T. The prevalence and determinant factors of self-reported depressive symptoms among elderly people with visual impairment attending an outpatient clinic in Ethiopia. Clinical optometry. 2021 Feb 17:63–72. doi: 10.2147/OPTO.S294618 33628068 PMC7898220

[44] Felker-Kantor EA, Wallace ME, Madkour AS, Duncan DT, et al. HIV stigma, mental health, and alcohol use disorders among people living with HIV/AIDS in New Orleans. Journal of urban health. 2019 Dec;96:878–88. doi: 10.1007/s11524-019-00390-0 31520231 PMC6904691

[45] Wardell JD, Shuper PA, Rourke SB, Hendershot CS. Stigma, coping, and alcohol use severity among people living with HIV: a prospective analysis of bidirectional and mediated associations. Annals of Behavioral Medicine. 2018 Sep;52(9):762–72. doi: 10.1093/abm/kax050 30124756 PMC6128374

[46] Tanzania national guidelines for the management of HIV and AIDS, 7^th^ edition April 2019.

[47] Kekwaletswe CT, Morojele NK. Patterns and predictors of antiretroviral therapy use among alcohol drinkers at HIV clinics in Tshwane, South Africa. AIDS care. 2014 Jul 4;26(sup1):S78–82. doi: 10.1080/09540121.2014.906558 24731102

